# Long-term outdoor air pollution and risk of ovarian and endometrial cancers in a large prospective cohort

**DOI:** 10.1038/s41370-026-00901-7

**Published:** 2026-06-15

**Authors:** Samantha Ammons, Jared A. Fisher, Jessica M. Madrigal, Lauren M. Hurwitz, Joel D. Kaufman, Alexandra J. White, Britton Trabert, Debra T. Silverman, Katie M. Applebaum, Megan A. Clarke, Rena R. Jones

**Affiliations:** 1 Occupational and Environmental Epidemiology Branch, Division of Cancer Epidemiology and Genetics (DCEG), National Cancer Institute (NCI), National Institutes of Health (NIH), Rockville, MD USA; 2 Department of Environmental and Occupational Health Sciences, University of Washington, Seattle, WA USA; 3 Epidemiology Branch, Chronic Disease Epidemiology Group, National Institute of Environmental Health Sciences, National Institutes of Health, Durham, NC USA; 4 Department of Obstetrics and Gynecology, Fox Spencer Eccles School of Medicine, University of Utah, and Huntsman Cancer Institute at the University of Utah, Salt Lake City, UT USA; 5 Department of Environmental and Occupational Health, The George Washington University, Milken Institute School of Public Health, Washington, DC USA; 6 Clinical Genetics Branch, DCEG, NCI, NIH, Rockville, MD USA; 7 AbbVie Inc, North Chicago, IL USA

**Keywords:** Ambient air pollution, Endometrial cancer, Ovarian cancer, PM_2.5_, NO_2_

## Abstract

**Background:**

Outdoor air pollution is a known human carcinogen, but studies of female reproductive malignancies other than breast cancer remain limited.

**Objective:**

We evaluated associations between outdoor air pollutants and risk of two gynecologic cancers

**Methods:**

Using nationwide spatiotemporal models, we estimated outdoor residential fine particulate matter (PM_2.5_, < 2.5 µm) and nitrogen dioxide (NO_2_) concentrations for 140,457 female NIH-AARP Diet and Health Study participants (enrolled 1995–1996), with follow-up through 2018. We used Cox regression to estimate hazard ratios (HR) and 95% confidence intervals (CIs) for associations between time-varying, 5-year average PM_2.5_ and NO_2_ concentrations (per 5 µg/m^3^ and ppb, respectively; 1980–2017) and incident endometrial (*n* = 3088) and ovarian (*n* = 1582) cancers, overall and by histologic subtype. Models were adjusted for age and demographic, lifestyle, and neighborhood factors. We evaluated co-pollutant adjustment and effect modification by state (per state-specific interquartile ranges), neighborhood deprivation, and body mass index (BMI).

**Results:**

We found no relationships with risk of either cancer overall. PM_2.5_ was associated with endometrial cancer among participants in Atlanta, Georgia (HR:1.70, CI = 1.16–2.48) and Pennsylvania (HR:1.15, CI = 1.02–1.29). A weaker NO_2_ association was apparent in these areas (HR:1.57, CI = 1.11–2.22 and HR:1.10, CI = 0.94–1.28, respectively). Associations with endometrial cancer were suggested in the highest quintile of neighborhood deprivation (HR PM_2.5_: 1.05; CI = 0.90–1.22; p-trend=0.07) and HR NO_2_:1.06, CI = 1.00–1.12; p-trend=0.02. We found positive, albeit imprecise, ovarian cancer associations in Detroit, Michigan (PM_2.5_ HR:1.42, CI = 0.92–2.18 and NO_2_ HR:1.72, CI = 0.95–3.13). NO_2_ was positively associated with non-endometrioid endometrial cancers (HR:1.12, CI = 0.94–1.35) and PM_2.5_ with non-epithelial ovarian tumors (HR:1.50, CI = 0.91–2.47). The impact of co-pollutant adjustment varied across state-specific associations. Relationships did not vary by BMI.

**Significance:**

We demonstrated modest relationships between air pollutants and risk of endometrial and ovarian cancers for select geographies, neighborhood characteristics, and histologic subtypes. Our findings indicate that future studies should evaluate region-specific factors, including co-varying pollutant distributions.

**Impact Statement:**

Research on endometrial and ovarian cancers has primarily focused on risk factors such as body mass index and exposure to exogenous and endogenous hormones, particularly unopposed estrogen. We identified suggestive associations between these two cancers and outdoor air pollution exposures, specifically PM_2.5_ and NO_2,_ in specific geographic regions and for certain histologic subtypes. Our novel findings suggest that PM_2.5_ chemical constituency and the region-specific air pollution mixture may play a role in the development of these cancers and warrants future investigation.

## Introduction

Endometrial and ovarian cancers are the first and second most common female gynecologic cancers diagnosed in the U.S., respectively [1], with notable racial and ethnic and socioeconomic disparities in incidence, stage at diagnosis, and survival [2]. Research on these malignancies has primarily focused on modifiable risk factors including elevated body mass index (BMI) and exposure to exogenous and endogenous hormones, particularly unopposed estrogen [3] as well as chronic inflammation [4, 5]. However, exposure to outdoor air pollutants, which arise from both anthropogenic and natural sources including traffic, may also relate to these cancers through diverse mechanisms. For example, fine particulate matter (< 2.5 µm, PM_2.5_) and nitrogen dioxide (NO_2_) have been shown to induce oxidative stress, promote chronic inflammation, and damage DNA [6, 7], supporting the biologic plausibility of associations with cancers at multiple organ sites. Additionally, some air pollution components are recognized as endocrine disruptors and can alter the function of the endocrine system including estrogen signaling pathways, potentially contributing to risk of estrogen-dependent malignancies, especially endometrial cancer [8]. While the biologic mechanisms are likely complex, air pollution has been shown to have obesogenic impacts by increasing inflammation and oxidative stress in adipose tissue [9]. Consequently, exposure to air pollution may have an impact on obesity-related malignancies such as endometrial and (to a lesser degree) ovarian cancers [10]. Moreover, individual pollutants like PM_2.5_ and NO_2_ may act through both shared and distinct biological pathways to initiate cancers.

Outdoor air pollution is classified by the International Agency for Research on Cancer (IARC) as a human carcinogen primarily based on evidence that exposure causes lung cancer [11]. Despite plausibility, studies of associations with ovarian and endometrial cancers remains limited. PM_2.5_ and NO_2_ are widespread ambient air pollutants that have been associated with increased ovarian [12] and endometrial [13] cancer risk or mortality in a small number of studies, although findings for ovarian cancer have been inconsistent [14], and studies of endometrial cancer are comparatively sparse but also with mixed findings [13, 15]. Few of the prior studies of these relationships evaluated time-varying exposures or heterogeneity in associations by tumor histologic subtype. We aimed to investigate whether exposure to outdoor PM_2.5_ and NO_2_ independently increase the risk of these cancers in a large, geographically spread cohort with long-term exposure assessment.

## Materials and methods

### Study population

We used data from the NIH-AARP Diet and Health Study, a prospective cohort initiated when 566,398 members of the AARP (formerly the American Association of Retired Persons) responded to a mailed questionnaire in 1995–1996. Participants were 50-71 years old at enrollment and recruited from six U.S. states (California, Florida, Louisiana, New Jersey, North Carolina, and Pennsylvania) and two metropolitan areas (Atlanta, Georgia, and Detroit, Michigan). The enrollment questionnaire included demographic and anthropometric characteristics, health-related factors, and dietary intakes. Information on additional cancer risk factors, including some reproductive history, was collected in a subsequent questionnaire for a subset of female participants (65%) in 1996–1997 [16]. Additional details on the study design and methodology are described elsewhere [17]. All participants provided written informed consent. The study was approved by the National Institutes of Health Institutional Review Board and the NIH-AARP Steering Committee.

Among the cohort’s women (*N* = 226,732), we excluded individuals who had a proxy respondent complete their survey (*N* = 1265) or had a cancer diagnosis prior to study enrollment (*N* = 23,390) and then drew independent subsets for the analyses of ovarian and endometrial cancers from the remaining 202,077 women. For the ovarian cancer analytic cohort, we further excluded women who were found to have died of ovarian cancer during follow-up, but the diagnosis was not confirmed in the registries (*N* = 482), had an ovarian cancer diagnosis prior to enrollment (*N* = 13), self-reported having a bilateral oophorectomy, other surgery, or unknown ovary status before enrollment (*N* = 61,063), had missing neighborhood level socioeconomic deprivation index (NDI) data (*N* = 165), missing air pollution data (*N* = 65), or for whom person-time could not be estimated (*N* = 61). For the endometrial cancer analysis, we excluded women who died of endometrial cancer but the diagnosis was not confirmed by registry (*N* = 184), were diagnosed prior to enrollment (*N* = 56), self-reported having a hysterectomy or did not know hysterectomy status before enrollment (*N* = 85,211), had missing NDI data (*N* = 130), missing air pollution data (*N* = 62), or for whom person-time could not be estimated (*N* = 54). The ovarian cancer analysis subset included 140,228 participants and 1582 cases, and the endometrial cancer analysis included 116,380 participants and 3088 cases.

### Cancer ascertainment

Diagnoses of ovarian and endometrial cancers were identified from probabilistic linkages with state-wide cancer registries in the catchment states and the three states to which participants most frequently moved (Arizona, Nevada, and Texas). Registry information included diagnosis date, grade, morphology, and stage. Ovarian cancer was classified according to the International Classification of Diseases for Oncology (ICD-O; third edition) site codes: C56.9 for ovary, C57.0 for fallopian tube, and C48.1 and C48.2 for peritoneum [18]. Endometrial cancer was classified using codes: C54.0, C54.1, C54.2, C54.3, C54.8, C54.9, and C55.9 [18]. Histology information was unknown for *N* = 48 (3%) of ovarian and *N* = 77 (2.5%) of endometrial cancer cases. Person-years of follow-up were calculated from the date the baseline questionnaire was received until whichever came first: the date that participants were diagnosed with ovarian or endometrial cancer, died, moved out of a registry ascertainment area, were lost to contact tracing, or the end of the follow-up period (December 31, 2018).

### Exposure assessment

Using previously-validated spatiotemporal models, annual exposures to ambient air pollutants were estimated at the participant enrollment addresses from 1980 to 2017 for PM_2.5_ and 1990 to 2017 for NO_2_ [19–21]. PM_2.5_ and NO_2_ models were developed independently but used the same overall framework, relying on a combination of data from regulatory and non-regulatory monitoring networks as well as satellite-derived measurements. To estimate PM_2.5_ concentrations prior to the widespread availability of monitoring data, linear back-extrapolation was conducted using temporal trends derived from monitoring data spanning 1999–2010 [20]. We did not have complete residential histories for participants and assumed women were living at their enrollment address throughout the exposure period. However, through tracing efforts, we were able to assess whether participants had moved from the start of the study through 2012, approximately 17 years after enrollment.

### Statistical analysis

We evaluated population demographic characteristics by PM_2.5_ and NO_2_ concentration in quartiles (using the ovarian cancer analysis group). We used Cox proportional hazards models with calendar time as the time variable to estimate hazard ratios (HRs) and 95% confidence intervals (CIs) of the association between five year moving average exposure to PM_2.5_ and NO_2_ and incident ovarian and endometrial cancers. The risk sets were defined based on two-year intervals throughout the follow-up period. For each risk set, we estimated a new five-year average for all participants remaining in the set (including cases diagnosed in this interval and all other non-censored participants). We parameterized the time-varying pollutant concentrations as a continuous variable (per each 5 µg/m^3^ and ppb increase in PM_2.5_ and NO_2_) and in quartiles (with the reference group being the lowest exposure category), evaluating exposure-response by parameterizing the median of each quartile as a continuous variable and assessing a test for trend (*p*-trend). Additionally, since the latency period for both cancers is estimated to be at least 10–20 years, we evaluated 5-year moving average exposures lagged 10 years for PM_2.5_ and 5 years for NO_2_ (since NO_2_ data was not available before 1990). We also conducted a sensitivity analysis using fixed 5-year historical time periods: 1980–1984 (PM_2.5_ only), 1985–1989 (PM_2.5_ only), and 1990–1994 (PM_2.5_ and NO_2_) to evaluate consistency with the main exposure parameterization. For state-specific analyses, we evaluated associations with continuous exposures with a unit change based on the respective state-specific interquartile ranges for PM_2.5_ and NO_2_. We conducted sensitivity analyses limited to participants with address geocodes at an exact address or street intersection level (i.e., good geocodes; *N* = 106,828; 91.8%), and separately after excluding women known to have moved between enrollment through 2012.

We used a literature-based approach to select potential confounders, identifying factors associated with ovarian and endometrial cancer and PM_2.5_ and/or NO_2_ exposure. From this list, we explored possible causal pathways and derived a minimal sufficient set of adjustment variables using a directed acyclic graph (Figs. S1–S2). Because information on some reproductive risk factors was available only for a subset of women, we separately evaluated exclusion of these factors (ever/never oral contraceptive use and ever/never menopausal hormone therapy use) from models and found limited influence on associations (< 1% change in HRs). Our final model covariates included age (continuous), race and ethnicity (Non-Hispanic White, Non-Hispanic Black, Hispanic, Asian, Pacific Islander, or American Indian/Alaska Native), smoking status and intensity [never smokers, former smokers (< 1 pack a day), former smokers (1–2 packs a day), former smokers (2+ packs a day), current smokers or just quit (< 1 pack a day), current smokers or just quit (1–2 packs a day), current smoker or just quit (2+ packs a day), or missing], neighborhood level socioeconomic deprivation index (NDI; continuous) [22], and state of residence (Model 1). Endometrial cancer analyses were also adjusted for BMI (< 25, 25–29, ≥30.0 kg/m^2^) since obesity is a major risk factor [23]. Covariate information was missing for ≤3% of participants; therefore, we coded unknown/missing as a separate category for analysis. Since parity is associated with both ovarian and endometrial cancers [24, 25], we conducted a sensitivity analysis with parity in the model. We also evaluated main effects associations with PM_2.5_ and NO_2_ in mutually adjusted models (Model 2). PM_2.5_ and NO_2_ concentrations were positively correlated for the 5-year average prior to enrollment, 1990–1994 (Spearman’s rho=0.73), so we also separately evaluated overall associations in cross-classified models using a common referent group of low concentrations (1^st^ tertile) of both pollutants. All statistical analyses were performed in SAS (v. 9.4).

We evaluated associations stratified by potential effect modifiers, including BMI and state of residence, since the distribution and chemical composition of PM_2.5_ varies by region [26], and to address potentially important, but unmeasured, spatially varying confounders that vary by region. We also stratified by the NDI, a census-tract level index generated previously for the NIH-AARP cohort [22], given the relationship in the U.S. between low socioeconomic status (SES) and higher exposure to ambient air pollutants [27]. We analyzed the NDI scores in quintiles, with the lowest quintile representing the ‘least deprived’ and highest representing the “most deprived”.  Additionally, we stratified by smoking status (never, former, and current smoker), given evidence of an inverse relationship with endometrial cancer [28, 29]. We evaluated heterogeneity (p-heterogeneity) in associations by histologic subtype using Cochran’s Q statistic in R. We evaluated statistical interactions using a joint Cox and Wald tests (*p*-interaction) for all other stratified analyses. The threshold for statistical significance for all analyses was *p* < 0.05.

## Results

Baseline lifestyle and demographic characteristics in the endometrial cancer analysis subset were similar across PM_2.5_ quartiles. The mean age at baseline was approximately 62 years and 90% of participants were non-Hispanic White. Within the highest category of outdoor residential PM_2.5_ levels, nearly a third of participants resided in an area ranked in the top quintile of neighborhood deprivation (Table 1). Patterns in these characteristics were similar among women in the ovarian cancer analyses (Table S1) and across NO_2_ quartiles (Tables S2 and S3).

**Table 1:** Baseline characteristics of NIH-AARP Diet and Health Study female participants included in the analyses of endometrial cancer, by historical outdoor PM_2.5_ levels at the residence^a,b^.

Characteristics	All participants	PM_2.5_ concentration (1990–1994; μg/m^3^)			
		Q1: 3.5–13.3	Q2: 13.4–15.6	Q3: 15.7–17.7	Q4: 17.8–27.0
**Participants (N)**	116,380	29,087	29,092	29,105	29,096
**NO**_**2**_**, ppb (mean ± SD)**^**c**^	16.0 ± 9.1	8.9 ± 3.6	12.9 ± 5.4	16.9 ± 5.5	25.3 ± 10.1
**Age (mean ± SD)**	61.7 ± 5.5	62.1 ± 5.5	61.6 ± 5.5	61.5 ± 5.5	61.6 ± 5.5
**Age groups (%)**					
50–54 years	15.8	14.5	16.3	16.6	16.0
55–59 years	23.3	21.5	23.9	23.9	24.0
60–64 years	27.2	27.5	27.0	27.1	27.1
65–69 years	30.3	32.9	29.5	29.3	29.7
≥70 years	3.4	3.6	3.5	3.1	3.3
**Race and ethnicity (%)**					
Non-Hispanic White	90.3	93.8	92.5	91.4	83.7
Non-Hispanic Black	4.7	1.9	2.9	4.7	9.4
Hispanic	1.9	2.2	1.6	1.1	2.6
Asian, PI, or AI/AK	1.8	0.9	2.0	1.6	2.6
Unknown	1.3	1.1	1.2	1.2	1.7
**Education level (%)**					
less than high school	5.5	5.6	5.2	5.3	5.7
completed high school	24.7	24.3	23.9	25.5	24.9
post high school	10.0	11.1	10.2	9.6	9.2
some college	24.0	25.7	23.6	22.3	24.4
college and postgraduate	33.0	30.2	34.4	34.7	32.7
unknown	2.8	3.1	2.6	2.6	3.1
**BMI (mean ± SD)**^**d**^	26.7 ± 5.7	26.4 ± 5.5	26.6 ± 5.7	26.7 ± 5.7	27.0 ± 6.0
**BMI category (%)**^**e**^					
Underweight and normal	43.3	44.9	43.9	43.3	41.1
Overweight	31.5	31.9	31.3	31.4	31.4
Obese	21.8	19.9	21.5	22.0	23.9
Unknown	3.4	3.3	3.4	3.3	3.6
**Postmenopausal status (%)**					
Premenopausal	6.2	5.7	6.6	6.3	6.3
Postmenopausal	93.3	93.9	93.0	93.3	93.1
Unknown	0.5	0.5	0.4	0.5	0.6
**Smoking status (%)**					
Never smoker	43.8	40.7	44.7	45.2	44.5
Former smoker	36.8	38.7	37.2	35.9	35.4
Current smoker	16.5	17.7	15.3	16.0	17.0
Unknown	2.9	2.9	2.9	2.9	3.1
**State of residence (%)**					
California	31.8	24.4	32.4	22.7	47.7
Florida	20.0	63.0	15.6	1.5	0.0
Georgia (Atlanta)	2.6	0.0	0.2	2.8	7.3
Louisiana	2.7	1.9	5.2	3.7	0.0
Michigan (Detroit)	5.3	0.4	3.7	9.5	7.8
North Carolina	7.5	3.4	10.5	13.2	2.7
New Jersey	14.6	5.1	23.5	23.4	6.4
Pennsylvania	15.5	1.9	8.9	23.2	28.1
**Neighborhood Socioeconomic Deprivation Index Quintiles**					
Quintile 1 (least deprived)	–	9.5	25.3	26.1	16.5
Quintile 2	–	18.7	21.4	22.2	17.0
Quintile 3	–	26.5	18.3	16.9	18.1
Quintile 4	–	23.8	18.3	19.0	17.2
Quintile 5 (most deprived)	–	21.5	16.7	15.8	31.3

^a^Percentages may not add up to 100% due to rounding.

^b^Abbreviations: *PM* particulate matter, *SD* standard deviation, *BMI* body mass index, *PI* Pacific Islander, *AI/AK* American Indian/Alaska Native.

^c^Based on mean of annual values from 1990 to 1994.

^d^*n* = 3968 women were missing BMI data and excluded from the mean and SD computation.

^e^Underweight and normal: BMI < 25.0 kg/m^2^; overweight: 25.0–29.9 kg/m^2^; obese: ≥30.0 kg/m^2^.

We observed no association between PM_2.5_ and risk of endometrial cancer overall with or without NO_2_ adjustment (Table 2). In stratified analyses, participants in Atlanta, Georgia, had a 1.7-fold increase in endometrial cancer risk associated with PM_2.5_ (*n* = 81 cases, HR per 2.0 µg/m^3^: HR = 1.70, CI = 1.16–2.48) and a positive association was also observed among women in Pennsylvania (*n* = 523 cases; HR per 2.2 µg/m^3^ = 1.15; CI = 1.02–1.29; Table 3). An elevated risk of PM_2.5_-associated endometrial cancer was apparent for women in the highest quintile of neighborhood deprivation (Q5 HR = 1.05; CI = 0.90–1.22; p-interaction = 0.07; Table 3). When state-specific PM_2.5_ models were mutually adjusted for NO_2_, patterns of association were similar for residents in Atlanta (HR per 2 µg/m^3^ = 1.52, CI = 0.85–2.73) and Pennsylvania (HR per 2 µg/m^3^ = 1.15, CI = 1.01–1.32). Participants in Atlanta had a 1.5-fold increased risk of endometrial cancer associated with NO_2_ (*n* = 81 cases; HR per 5 ppb=1.52; CI = 1.10–2.09; Table 4) and the association was attenuated after PM_2.5_ adjustment (HR per 5 ppb = 1.14, CI = 0.67–1.92; Table 4). The NO_2_-endometrial cancer association was strongest among those living in the most deprived neighborhoods (Q5 HR = 1.06, CI = 1.00–1.12; p-interaction = 0.02; Table 4). We found no evidence of interaction by BMI category on endometrial cancer associations with either pollutant (Tables 3, 4). Associations with PM_2.5_ and NO_2_ for the ‘good geocode’ and parity-adjusted sensitivity analyses were consistent with our findings in the main analysis (changes in HRs <5%). We observed similar null associations in main effects for both cancers across fixed 5-year historical time periods (changes in HRs <5%) as with the models of time-varying exposures (Table S4).

**Table 2:** Associations between long-term residential PM_2.5_ and NO_2_ concentrations and risk of endometrial and ovarian cancers in the NIH-AARP cohort^a^.

Endometrial cancer						Ovarian cancer	
**PM**_**2.5**_	**Cases**	**HR (95% CI)** **Model 1**^**b**^	**HR (95% CI)** **Model 2**^**c**^		**Cases**	**HR (95% CI)** **Model 1**^**b**^	**HR (95% CI)** **Model 2**^**c**^
Per 5 μg/m^3^	3088	1.00 (0.92–1.08)	0.97 (0.87–1.08)	Per 5 μg/m^3^	1582	0.94 (0.84–1.05)	0.93 (0.79–1.08)
Q1	719	1.00 (referent)	1.00 (referent)	Q1	400	1.00 (referent)	1.00 (referent)
Q2	782	0.97 (0.86–1.10)	0.98 (0.86–1.11)	Q2	405	1.00 (0.85–1.17)	0.99 (0.84–1.17)
Q3	777	0.97 (0.86–1.11)	0.98 (0.84–1.14)	Q3	381	0.95 (0.79–1.13)	0.94 (0.77–1.15)
Q4	810	0.99 (0.87–1.13)	1.00 (0.85–1.17)	Q4	396	0.94 (0.79–1.13)	0.94 (0.75–1.17)
*p* for trend		0.90	0.90			0.67	0.85
**NO**_**2**_	**Cases**	**HR (95% CI)** **Model 1**^**b**^	**HR (95% CI)** **Model 2**^**c**^		**Cases**	**HR (95% CI)** **Model 1**^**b**^	**HR (95% CI)** **Model 2**^**c**^
Per 5 ppb	3088	1.01 (0.98–1.04)	1.02 (0.97–1.06)	Per 5 ppb	1582	0.99 (0.95–1.03)	1.01 (0.95–1.07)
Q1	751	1.00 (referent)	1.00 (referent)	Q1	408	1.00 (referent)	1.00 (referent)
Q2	730	0.96 (0.87–1.07)	0.96 (0.86–1.07)	Q2	370	0.92 (0.79–1.06)	0.93 (0.80–1.08)
Q3	786	0.98 (0.88–1.09)	0.98 (0.87–1.11)	Q3	415	1.01 (0.87–1.17)	1.03 (0.88–1.22)
Q4	821	0.99 (0.88–1.11)	0.99 (0.86–1.14)	Q4	389	0.94 (0.80–1.11)	0.98 (0.80–1.21)
*p* for trend		0.90	0.90			0.57	0.90

^a^Abbreviations: *PM* particulate matter, *NO*_*2*_ nitrogen dioxide, *HR* hazard ratio, *CI* confidence interval, *μg/m*^*3*^ micrograms per cubic meter, *ppb* parts per billion.

^b^Adjusted for age, race and ethnicity, smoking status and intensity, neighborhood socioeconomic deprivation index, body mass index (endometrial cancer only), and state of residence.

^c^Adjusted for Model 1 covariates and mutually adjusted for either NO_2_ or PM_2.5_.

**Table 3:** Association between long-term residential PM_2.5_ concentrations (per 5 μg/m^3^) and risk of endometrial and ovarian cancers in the NIH-AARP cohort, by state, BMI category, and NDI quintiles^a^.

	Endometrial cancer (*N* = 116,380)				Ovarian cancer (*N* = 140,228)			
	Cases	Mean ± SD (μg/m^3^)^b^	HR (95% CI) Model 1^c^	HR (95% CI) Model 2 ^d^	Cases	Mean ± SD (μg/m^3^)^b^	HR (95% CI) Model 1^c^	HR (95% CI) Model 2 ^d^
**State of residence**^**e**^								
California	1030	19.7 ± 4.5	0.99 (0.90–1.10)	0.96 (0.82–1.13)	570	19.7 ± 4.5	0.88 (0.77–1.01)	0.82 (0.66–1.02)
Florida	520	15.1 ± 1.8	0.92 (0.83–1.03)	0.94 (0.84–1.06)	294	15.1 ± 1.8	1.04 (0.90–1.21)	1.06 (0.91–1.24)
Georgia (Atlanta)	81	21.8 ± 1.7	1.70 (1.16–2.48)	1.52 (0.85–2.73)	46	21.7 ± 1.7	1.06 (0.65–1.71)	1.03 (0.47–2.27)
Louisiana	92	17.8 ± 2.0	1.02 (0.75–1.37)	0.80 (0.51–1.26)	58	17.8 ± 2.0	0.95 (0.64–1.40)	1.10 (0.61–1.99)
Michigan (Detroit)	151	20.5 ± 2.0	0.92 (0.71–1.18)	0.82 (0.57–1.19)	62	20.6 ± 2.0	1.42 (0.92–2.18)	1.18 (0.65–2.13)
North Carolina	194	18.6 ± 2.3	0.89 (0.73–1.09)	0.84 (0.63–1.12)	123	18.6 ± 2.3	0.92 (0.72–1.18)	0.80 (0.56–1.13)
New Jersey	497	19.0 ± 2.2	1.01 (0.88–1.15)	1.01 (0.83–1.24)	189	19.0 ± 2.2	0.97 (0.78–1.21)	1.01 (0.73–1.41)
Pennsylvania	523	20.6 ± 2.1	1.15 (1.02–1.29)	1.17 (1.01–1.35)	240	20.6 ± 2.1	1.20 (1.01–1.42)	1.21 (0.98–1.49)
p-interaction			0.71	0.72			0.67	0.65
**BMI category**^**f**^								
Underweight and normal	840	18.7 ± 3.6	1.02 (0.88–1.18)	0.96 (0.78–1.19)	696	18.6 ± 3.6	0.92 (0.79–1.09)	0.92 (0.73–1.17)
Overweight	904	18.8 ± 3.6	1.04 (0.90–1.21)	0.93 (0.75–1.15)	480	18.7 ± 3.6	0.94 (0.77–1.14)	0.80 (0.60–1.07)
Obese	1260	19.0 ± 3.6	0.97 (0.86–1.09)	1.01 (0.85–1.19)	356	19.0 ± 3.7	0.98 (0.79–1.22)	1.13 (0.82–1.54)
p-interaction			0.70	0.70			0.40	0.40
**Neighborhood Socioeconomic Deprivation Index (NDI) Quintiles**								
Q1 (least deprived)	595	19.2 ± 2.8	0.86 (0.69–1.07)	0.81 (0.60–1.09)	336	19.1 ± 2.9	0.95 (0.72–1.25)	0.83 (0.57–1.21)
Q2	596	18.7 ± 3.4	1.03 (0.86–1.23)	1.10 (0.83–1.45)	332	18.6 ± 3.4	0.88 (0.69–1.13)	0.84 (0.58–1.23)
Q3	621	18.3 ± 3.8	1.02 (0.87–1.19)	1.04 (0.82–1.31)	308	18.3 ± 3.7	0.96 (0.75–1.22)	1.14 (0.80–1.62)
Q4	611	18.3 ± 3.7	1.00 (0.84–1.18)	1.12 (0.88–1.44)	317	18.2 ± 3.9	0.93 (0.76–1.15)	0.89 (0.64–1.23)
Q5 (most deprived)	665	19.4 ± 4.1	1.05 (0.90–1.22)	0.89 (0.72–1.10)	289	19.5 ± 4.1	0.99 (0.78–1.25)	1.00 (0.73–1.38)
p-interaction			0.07	0.42			0.40	0.42
**Smoking Status**^**g**^								
Never Smoker	1629	15.8 ± 3.5	1.04 (0.94–1.16)	1.08 (0.93–1.26)	773	15.7 ± 3.2	0.84 (0.72–0.97)	0.96 (0.77–1.19)
Former Smoker	1105	15.5 ± 3.2	1.04 (0.91–1.17)	0.90 (0.75–1.08)	569	15.5 ± 3.2	1.11 (0.93–1.33)	1.03 (0.79–1.33)
Current Smoker	280	15.6 ± 3.3	0.76 (0.59–0.98)	0.66 (0.45–0.97)	199	15.6 ± 3.3	0.96 (0.71–1.30)	0.72 (0.46–1.13)
p-interaction			0.05	0.05			0.71	0.71

^a^Abbreviations: *PM* particulate matter, *HR* hazard ratio, *CI* confidence interval, *μg/m*^3^ micrograms per cubic meter.

^b^During 1990–1994.

^c^Adjusted for age, race and ethnicity, smoking status and intensity, neighborhood socioeconomic deprivation index, body mass index (endometrial cancer only), and state of residence.

^d^Adjusted for Model 1 covariates and mutually adjusted for either NO_2_ or PM_2.5_.

^e^State-specific continuous model associations are per the following state-specific PM_2.5_ interquartile range for: California: 6.3 μg/m^3^ ; Florida: 1.8 μg/m^3^; Atlanta, Georgia: 2.0 μg/m^3^; Louisiana: 2.2 μg/m^3^; Detroit, Michigan: 2.2 μg/m^3^; North Carolina: 2.6 μg/m^3^; New Jersey: 2.4 μg/m^3^; Pennsylvania: 2.2 μg/m^3^.

^f^*n* = 3968 women with unknown BMI excluded from the endometrial cancer analysis and *n* = 4713 from the ovarian cancer analysis.

^g^*n* = 3423 women with missing smoking status were excluded from the endometrial cancer analysis and *n* = 4113 women with missing smoking status were excluded from the ovarian cancer analysis.

**Table 4:** Association between long-term residential NO_2_ (per 5 ppb) concentrations and risk of endometrial and ovarian cancers in the NIH-AARP cohort, by state, BMI category, and NDI quintiles^a^.

	Endometrial cancer (*N* = 116,380)				Ovarian cancer (*N* = 140,228)			
	Cases	Mean ± SD (ppb)^b^	HR (95% CI) Model 1^c^	HR (95% CI) Model 2 ^d^	Cases	Mean ± SD (ppb)^b^	HR (95% CI) Model 1^c^	HR (95% CI) Model 2 ^d^
**State of residence**^**e**^								
California	1030	21.7 ± 11.2	1.01 (0.92–1.12)	1.04 (0.89–1.23)	570	21.7 ± 11.2	0.93 (0.80–1.08)	1.10 (0.87–1.39)
Florida	520	9.7 ± 3.6	0.90 (0.79–1.04)	0.93 (0.80–1.08)	294	9.7 ± 3.6	0.95 (0.78–1.16)	0.93 (0.75–1.14)
Georgia (Atlanta)	81	13.8 ± 4.2	1.57 (1.11–2.22)	1.15 (0.65–2.02)	46	13.7 ± 4.2	1.06 (0.63–1.79)	1.03 (0.44–2.44)
Louisiana	92	10.2 ± 5.0	1.23 (0.82–1.84)	1.54 (0.83–2.85)	58	10.4 ± 5.0	0.83 (0.48–1.45)	0.75 (0.33–1.74)
Michigan (Detroit)	151	15.1 ± 4.9	1.02 (0.71–1.47)	1.24 (0.74–2.09)	62	15.2 ± 4.9	1.72 (0.95–3.13)	1.47 (0.65–3.35)
North Carolina	194	8.9 ± 4.5	0.95 (0.73–1.24)	1.12 (0.76–1.65)	123	8.9 ± 4.5	1.05 (0.75–1.46)	1.31 (0.81–2.11)
New Jersey	497	17.5 ± 6.8	1.00 (0.86–1.16)	0.99 (0.79–1.24)	189	17.5 ± 6.8	0.95 (0.75–1.22)	0.94 (0.65–1.36)
Pennsylvania	523	16.0 ± 5.5	1.10 (0.94–1.28)	0.96 (0.79–1.17)	240	16.0 ± 5.5	1.14 (0.91–1.44)	0.98 (0.73–1.31)
p-interaction			0.35	0.32			0.76	0.79
**BMI category**^**f**^								
Underweight and normal	840	15.8 ± 8.9	1.01 (0.96–1.07)	1.02 (0.94–1.11)	696	15.8 ± 9.0	1.01 (0.95–1.06)	1.01 (0.93–1.09)
Overweight	904	15.9 ± 9.0	1.04 (0.98–1.09)	1.06 (0.98–1.14)	480	15.7 ± 9.1	1.04 (0.97–1.11)	1.09 (0.99–1.19)
Obese	1260	16.4 ± 9.2	0.99 (0.95–1.03)	0.99 (0.92–1.05)	356	16.3 ± 9.2	0.97 (0.89–1.04)	0.93 (0.84–1.04)
p-interaction			0.55	0.55			0.76	0.77
**Neighborhood Socioeconomic Deprivation Index Quintiles**								
Q1 (least deprived)	595	16.6 ± 7.5	0.98 (0.90–1.07)	1.03 (0.92–1.16)	336	16.6 ± 7.6	1.01 (0.92–1.11)	0.97 (0.86–1.10)
Q2	596	16.5 ± 8.6	1.00 (0.93–1.06)	0.97 (0.87–1.08)	332	16.4 ± 8.7	1.00 (0.92–1.09)	1.05 (0.93–1.19)
Q3	621	14.6 ± 9.2	1.00 (0.94–1.07)	0.99 (0.90–1.10)	308	15.3 ± 9.0	0.98 (0.90–1.07)	0.93 (0.83–1.05)
Q4	611	15.5 ± 8.9	0.97 (0.91–1.04)	0.94 (0.85–1.03)	317	14.4 ± 9.4	1.05 (0.97–1.14)	1.11 (0.99–1.24)
Q5 (most deprived)	665	16.9 ± 10.4	1.06 (1.00–1.12)	1.09 (1.01–1.18)	289	16.8 ± 10.3	1.00 (0.93–1.08)	1.02 (0.92–1.14)
p-interaction			0.02	0.02			0.42	0.40
**Smoking Status**^**g**^								
Never Smoker	1629	16.0 ± 9.2	1.00 (0.96–1.04)	0.98 (0.93–1.04)	773	15.8 ± 9.2	0.92 (0.86–0.98)	0.93 (0.85–1.02)
Former Smoker	1105	16.0 ± 8.9	1.05 (1.00–1.10)	1.08 (1.01–1.15)	569	15.9 ± 9.1	1.05 (0.98–1.13)	1.04 (0.95–1.15)
Current Smoker	280	16.0 ± 9.1	0.95 (0.87–1.05)	1.07 (0.93–1.23)	199	15.9 ± 9.1	1.06 (0.95–1.19)	1.16 (0.99–1.36)
p-interaction			0.12	0.12			0.13	0.13

^a^Abbreviations: *NO*_*2*_ nitrogen dioxide, *HR* hazard ratio *CI* confidence interval, ppb parts per billion.

^b^During 1990–1994.

^c^Adjusted for age, race and ethnicity, smoking status and intensity, neighborhood socioeconomic deprivation index, body mass index (endometrial cancer only), and state of residence.

^d^Adjusted for Model 1 covariates and mutually adjusted for either NO_2_ or PM_2.5_.

^e^State-specific continuous model associations are per the following state-specific NO_2_ interquartile range for: California: 14.0 ppb; Florida: 4.6 ppb; Atlanta, Georgia: 5.4 ppb; Louisiana: 7.8 ppb; Detroit, Michigan: 6.8 ppb; North Carolina: 6.5 ppb; New Jersey: 9.5 ppb; Pennsylvania: 7.6 ppb.

^f^*n* = 3968 women with unknown BMI excluded from the endometrial cancer analysis and *n* = 4713 from the ovarian cancer analysis.

^g^*n *= 3423 women with missing smoking status were excluded from the endometrial cancer analysis and *n* = 4113 women with missing smoking status were excluded from the ovarian cancer analysis.

We also observed no associations between either pollutant and risk of ovarian cancer overall (Table 2). Stratified analyses yielded a positive, although imprecise, PM_2.5_ association among participants in Detroit, Michigan (*n* = 62 cases; HR per 2.2 µg/m^3^: = 1.42; CI = 0.92–2.18) but no clear evidence of a relationship in other areas (p-interaction = 0.67; Table 3). The positive relationship with PM_2.5_ in Detroit was still apparent but weaker after adjustment for NO_2_ (HR per 2.2 µg/m^3^ = 1.18, CI = 0.65–2.13; Table 3). We did not find clear evidence that ovarian cancer associations with PM_2.5_ or NO_2_ varied across strata of BMI or neighborhood deprivation (p-interaction >0.05; Tables 3, 4). We found no relationship between PM_2.5_ and endometrial cancer among never and former smokers, although an inverse association was apparent in current smokers (HR per 5 μg/m^3^ = 0.76; CI = 0.59–0.98) and persisted with NO_2_ adjustment (HR = 0.66; CI = 0.45–0.97; p-interaction = 0.05; Table 3). This pattern was not evident for ovarian cancer. We observed some positive associations for NO_2_ across smoking strata for both cancer sites, but for most, neither the point estimates nor the interaction was statistically significant (p-interaction ≥0.05; Table 4).

Analyses by histologic type yielded few statistically significant associations and no evidence of heterogeneity (p-heterogeneity>0.05) but suggested some positive relationships (Table 5). Risk of non-endometroid endometrial cancers was elevated in association with PM_2.5_ (HR per 5 µg/m^3^: 1.12; CI = 0.94–1.35); the positive relationship with PM_2.5_ was still apparent after adjustment for NO_2_ (HR per 5 µg/m^3^ = 1.08, CI = 0.84–1.39; Table 5). Risk of non-endometrioid endometrial cancers was also elevated for NO_2_ exposure (HR per 5 ppb=1.05, CI = 0.98–1.12; Table 5) and we found no association with endometrioid or other types. Non-epithelial ovarian tumors were positively associated with PM_2.5_ (HR = 1.50, CI = 0.91–2.47) and a weaker but positive association was apparent for NO_2_ (HR = 1.05, CI = 0.87–1.27).

**Table 5:** Associations between long-term residential PM_2.5_ and NO_2_ concentrations and risk of endometrial and ovarian cancer histologic subtypes in the NIH-AARP cohort.

PM_2.5_ (per 5 μg/m^3^)				
Endometrial cancer (*N* = 116,380)				
	Cases	Mean ± SD (μg/m^3^)^a^	HR (95% CI) Model 1^b^	HR (95% CI) Model 2^c^
Endometrioid	2368	15.7 ± 3.3	0.98 (0.90–1.07)	0.96 (0.85–1.09)
Non-Endometrioid	583	16.2 ± 3.3	1.12 (0.94–1.35)	1.08 (0.84–1.39)
Other	60	15.6 ± 3.2	0.71 (0.41–1.26)	0.78 (0.34–1.75)
p-heterogeneity			0.21	0.61
**Ovarian cancer** (***N*** = **140,228)**				
	**Cases**	**Mean ± SD (μg/m**^**3**^**)**^**a**^	**HR (95% CI)** **Model 1**^**b**^	**HR (95% CI)** **Model 2**^**c**^
Serous	729	15.6 ± 3.3	0.92 (0.79–1.07)	0.84 (0.67–1.06)
Non-Serous	605	15.7 ± 3.2	0.95 (0.80–1.13)	0.97 (0.76–1.25)
Non-Epithelial	75	16.1 ± 3.2	1.50 (0.91–2.47)	1.86 (0.93-3.69)
Other	125	15.4 ± 3.6	0.72 (0.47–1.11)	0.61 (0.33–1.14)
p-heterogeneity			0.18	0.09
**NO**_**2**_ **(per 5 ppb)**				
**Endometrial cancer** (***N*** = **116,380)**				
	**Cases**	**Mean ± SD (ppb)**^**a**^	**HR (95% CI)** **Model 1**^**b**^	**HR (95% CI)** **Model 2**^**c**^
Endometrioid	2368	16.2 ± 9.2	1.00 (0.97–1.03)	1.01 (0.96–1.06)
Non-Endometrioid	583	17.2 ± 9.7	1.05 (0.98–1.12)	1.02 (0.93–1.13)
Other	60	14.8 ± 9.1	0.88 (0.70–1.12)	0.95 (0.68–1.34)
p-heterogeneity			0.23	0.92
**Ovarian cancer** (***N*** = **140,228)**				
	**Cases**	**Mean ± SD (ppb)**^**a**^	**HR (95% CI)** **Model 1**^**b**^	**HR (95% CI)** **Model 2**^**c**^
Serous	729	16.1 ± 9.1	1.00 (0.94–1.06)	1.05 (0.96–1.15)
Non-Serous	605	15.8 ± 8.8	0.98 (0.92–1.05)	0.99 (0.90–1.09)
Non-Epithelial	75	16.4 ± 9.9	1.05 (0.87–1.27)	0.89 (0.67–1.17)
Other	125	15.6 ± 7.7	0.91 (0.78–1.06)	1.10 (0.85–1.42)
p-heterogeneity			0.63	0.57

^a^During 1990–1994.

^b^Adjusted for age, race and ethnicity, smoking status and intensity, neighborhood socioeconomic deprivation index, body mass index (endometrial cancer only), and state of residence.

^c^Adjusted for Model 1 covariates and mutually adjusted for either NO_2_ or PM_2.5_.

As in the main analysis, we also observed no association for either exposure and either cancer after excluding women known to have moved between enrollment and first follow-up (Table S5). We also observed no association in our lagged analyses, with or without mutual adjustment (Table S6). Lastly, we found no evidence of associations when levels of PM_2.5_ were high and NO_2_ levels were low (and vice versa) when using a common referent approach. Moreover, the associations for both cancers were null regardless of the relative levels of either pollutant compared to a common referent group of low exposure to both pollutants (Table S7).

## Discussion

In this large prospective study, we observed no overall association between long-term outdoor PM_2.5_ or NO_2_ concentrations at the residence and risk of either endometrial or ovarian cancer. However, associations with PM_2.5_ and NO_2_ for both cancers were apparent in select geographic areas, and endometrial cancer risk was elevated in the most deprived neighborhoods (highest quintile of the NDI), with a stronger association observed for NO_2_. We also found some evidence that associations with PM_2.5_ and NO_2_ differed across endometrial and ovarian cancer histologic subtypes.

Our finding of no overall association between PM_2.5_ and endometrial cancer is consistent with recent work in the Sister Study, which also found no relationship [13]. The Sister Study did however report a positive endometrial cancer association with NO_2_, a weakly genotoxic and mutagenic pollutant [30, 31] often considered a marker for vehicular traffic and its carcinogenic constituents, such as benzene. We did not observe an overall endometrial cancer association with NO_2_ in our analysis. PM_2.5_ concentrations in our cohort were higher median (IQR): 18.79 (16.1–21.2 μg/m^3^) than in the Sister Study (10.7) (8.7–12.4 μg/m^3^), although NO_2_ levels were more comparable: 7.6 (5.1–10.8ppb) versus 8.5 (5.8–11.9 ppb). Possible explanations for the differences in findings may be attributed to differing population characteristics, including demographics (the AARP cohort is older, with a mean age of 62 years versus 54 in the Sister Study), geographic coverage (our 8 catchment areas versus nationwide in the Sister Study), and the proportion of post-menopausal women ( > 90% versus ~75%). The exposure periods of the studies were also different; the Sister Study exposures covered a prospective period 2003–2017, whereas in the NIH-AARP cohort spanned a 35+ year period (1980–2017) that included 15 years prior to enrollment.

Our analyses did yield a positive PM_2.5_ association with endometrial cancer among women residing in select geographic areas. Specifically, the relationship was evident among participants residing in Atlanta and Pennsylvania, for which there are several potential explanations. Mean residential levels of PM_2.5_ were among the highest for these participants across our catchment areas, although we did not find a similar positive association among women living in other areas with similarly high concentrations (e.g., California and Detroit). This could indicate that varying PM_2.5_ chemical constituency, resulting from its diverse sources or other regional factors, may be driving the observed relationships. For example, PM_2.5_ is comprised of a higher percentage of sulfate in eastern counties compared to western counties in the U.S [32]. Additionally, one study found that long-term exposure to specific constituents of PM_2.5_, nickel, and vanadium (chemicals that are commonly found in mixed oil burning industrial emissions) were associated with cancer of the upper aerodigestive tract after adjustment for NO_2_ exposure [33]. Moreover, three prior cancer analyses conducted within the NIH-AARP cohort have also reported regional heterogeneity in the PM_2.5_-cancer associations, including for incident breast [34], liver [35], and lung [36] cancers. Therefore, it is possible that the chemical constituency in PM_2.5_ in our cohort differs regionally and are driving the differential associations between PM_2.5_ and endometrial cancer we observed by state.

We also found no evidence of an overall association between PM_2.5_ or NO_2_ and the development of ovarian cancer. The lack of finding for outdoor PM_2.5_ is consistent with a recent analysis in the Sister Study [12], which also found no association with ovarian cancer risk after adjustment for co-pollutants (NO_2_ and O_3_) and reproductive risk factors. The Sister Study reported a positive NO_2_ association with ovarian cancer, whereas we did not observe one. A recent systematic review found that exposure to ambient air pollutants (including but not limited to particulate matter such as PM_10_ and PM_2.5_, and gases such as sulfur dioxide and NO_2_) increased ovarian cancer risk in several studies conducted both within and outside of the U.S [14]. However, most of the reviewed studies were of ecological design. While we found no overall ovarian cancer relationship in the cohort, our subgroup analyses indicated associations with both pollutants. The strongest association with PM_2.5_ was observed among participants in Pennsylvania, and the finding persisted in mutually adjusted models. Consistent with this result, a weakly positive ovarian cancer association with NO_2_ in the Pennsylvania participants was diminished to the null after adjustment for PM_2.5_. Similarly, in our endometrial cancer findings for participants in Atlanta, the association attenuated once PM_2.5_ was added to the model. Collectively, these findings may implicate PM_2.5_, and not NO_2_, as a driver of the observed association with ovarian cancer in these states.

The protective association between PM_2.5_ exposure and endometrial cancer risk we observed in current smokers is interesting, though the mechanisms remain unclear. One possible explanation is that smoking may reduce cancer risk through antiestrogenic effects, such as altering estrogen metabolism and lowering circulating estrogen levels [37, 38], which could reduce the sensitivity of the endometrium to environmental pollutants like PM_2.5_. Given that our evaluation of this relationship was based only on smoking status at study enrollment and this is the first report of the potential interaction, these findings should be interpreted cautiously. Additionally, the lack of a similar pattern for NO_2_ further underscores the potentially complex nature of these associations.

A unique contribution of our work was the analyses by histologic subtype, for which findings may offer new insights about underlying etiologic pathways. We found an elevated risk of non-endometrioid endometrial cancers in our PM_2.5_ analysis and a similar, but weaker association with NO_2_. Non-endometrioid endometrial cancers are predominately serous and clear cell carcinoma, typically make up approximately 20% of all endometrial cancer cases, and have poorer prognosis than endometrioid carcinomas [23]. Risk factor associations for non-endometroid endometrial cancers are similar to endometrioid histologic subtypes, however, there is evidence that non-endometrioid carcinomas are not as estrogen dependent as endometrioid carcinomas [39]. Women with these non-endometrioid histologies tend to more frequently exhibit molecular and genetic alterations such as p53 mutations inactivation that play a role in cancer development [40], which we postulate may lead to increased vulnerability to the carcinogenic effects of PM_2.5_ and NO_2_. Our data also suggested increased risks of non-epithelial ovarian cancer associated with PM_2.5_. These cancers, which include ovarian sarcoma, malignant ovarian germ cell tumors, and sex cord-stromal tumors, make up roughly 10% of cases and are largely thought to be driven by genetic and developmental factors [41]. Although we observed a small increased risk of these tumors associated with PM_2.5_, the subtype heterogeneity complicates interpretations.

We stratified by BMI for several reasons. Obesity is an established risk factor for endometrial cancer [23] and is also associated with (though less strongly) ovarian cancer [42], and adipose tissue is a reservoir for lipophilic constituents of PM_2.5_, such as polycyclic aromatic hydrocarbons, that have been linked to carcinogenesis at multiple tumor sites [43]. Women with higher BMI may also be more vulnerable to the effects of PM_2.5_ exposure because overweight and obese individuals have increased levels of oxidated stress and inflammation, which are potential biological mechanisms for a PM_2.5_-endometrial cancer relationship [44]. Despite plausibility, our results did not indicate that the association with PM_2.5_ was stronger for overweight or obese women.

We observed that women living in the most highly deprived neighborhoods had an elevated risk of endometrial cancer associated with both PM_2.5_ and NO_2_, and more strongly so with NO_2_, whereas we found no associations among women in the least deprived neighborhoods. Lower socioeconomic status communities are more likely to be exposed to higher concentrations of environmental pollutants [45], a finding that aligns with our population, in which women with the highest levels of neighborhood deprivation represented the largest proportion (31%) of those with high (quartile 4) outdoor PM_2.5_ concentrations. In contrast to our findings, a recent analysis in the Sister Study found no clear pattern of uterine cancer association with either PM_2.5_ or NO_2_ by neighborhood socioeconomic status, as risks were either not increased or elevated non-monotonically across quartiles of an area deprivation index [13].

Our analysis included several strengths that advance the limited research on this topic. The NIH-AARP cohort includes large numbers of incident, well-characterized cancers. It is also geographically spread, with heterogeneity in air pollution exposures. We assessed PM_2.5_ and NO_2_ at individual participant residences rather than at the area level (e.g., census tract, ZIP code), as has been done in some prior studies. Additionally, our long-term, historical exposure assessment enabled time-varying analyses to account for secular trends in concentrations and the long latency of these solid tumors. A wide range of individual-level covariates were available for evaluation of effect modification and confounding. To our knowledge, our work reflects the first analysis of these associations by histologic subtype, an important approach to understanding potential underlying mechanisms.

Although exposures were assessed by validated models, these data still reflect estimates of concentrations around the home without accounting for time spent or exposures occurring in other microenvironments and thus may not represent personal exposures. We also based our exposure assessment on the enrollment address and had limited information on historical residence changes over time, which could have led to exposure misclassification. However, our findings were consistent in sensitivity analysis excluding women who were known to have moved between enrollment and 2012. We anticipate any resultant exposure misclassification to be non-differential with respect to cancer status, leading to potential attenuation of observed associations. We also acknowledge that our PM_2.5_ and NO_2_ estimates began in 1980 and 1990, respectively, which constrained the maximum possible lag period we could use for both pollutants. Potential confounders were also ascertained only at enrollment, and we acknowledge that we could not account for some changes in modifiable risk factors during follow-up. Our cross-classified analysis was consistent with our main effects in that we found no evidence of an overall association for PM_2.5_ or NO_2_ for either cancer. However, we were underpowered to use this approach for subgroup analyses to confirm our findings in mutually adjusted models. Additionally, the NIH-AARP cohort is predominantly non-Hispanic White, older, and drawn from eight catchment states. This demographic and geographic composition may limit the generalizability of our findings to more racially diverse or younger populations, and to other regions in the U.S. Finally, our ability to interrogate associations by histologic subtype was a study strength, but despite the large size of our cohort we acknowledge the small numbers of cases in these analyses and that associations were imprecise. Our findings should be interpreted cautiously due to low power to detect associations across some strata but, given limited prior study of these relationships, warrant further investigation.

## Conclusion

Our findings in this large cohort suggest that long-term exposure to PM_2.5_ and risk of endometrial cancer is limited primarily to specific geographic areas and potentially for women living in neighborhoods with the highest levels of deprivation. A weaker association was generally observed for NO_2_. Possible heterogeneity in associations by histologic subtype for both endometrial and ovarian cancers require replication. Collectively, the number of evaluations of these specific relationships is small and the findings remain equivocal. Future research that further evaluates pollutant constituency in large, more racially and ethnically diverse populations as well as studies that incorporate biomarkers and/or mechanistic models may help clarify these relationships.

## Supplementary information

Supplementary information

## Data Availability

Data are available upon approval of a proposal submitted to the NIH-AARP Diet and Health Study Steering Committee. For more information visit https://www.nihaarpstars.com/.
