# Supplementary material for: Long-term outdoor air pollution and risk of ovarian and endometrial cancers in a large prospective cohort
Source: J Expo Sci Environ Epidemiol. 2026 Jun 15;36(6):1194–204. doi: 10.1038/s41370-026-00901-7 (PMC13727682; doi:10.1038/s41370-026-00901-7)
Supplement: Supplementary file 1 [file 41370_2026_901_MOESM1_ESM.docx]

**Supplementary Information for**

**Long-term outdoor air pollution and risk of ovarian and endometrial cancers in a large prospective cohort**

Samantha Ammons, Jared A. Fisher, Jessica M. Madrigal, Lauren M. Hurwitz, Joel D. Kaufman, Alexandra J. White, Britton Trabert, Debra T. Silverman, Katie M. Applebaum, Megan A. Clarke, Rena R. Jones

**List of tables and figures**

**Table S1.** Baseline characteristics of NIH-AARP Diet and Health Study female participants included in the analyses of ovarian cancer, by historical outdoor PM_2.5_ levels at the residence

**Table S2.** Baseline characteristics of NIH-AARP Diet and Health Study population included in the analyses of endometrial cancer, by historical ambient NO_2_ levels at the residence

**Table S3.** Baseline characteristics of NIH-AARP Diet and Health Study population included in the analyses of ovarian cancer, by historical ambient NO_2_ levels at the residence

**Table S4**. Associations between 5-year historical average residential PM_2.5_ (µg/m^3^) and NO_2_ (ppb) concentrations and risk of endometrial and ovarian cancers in the NIH-AARP Diet and Health Study

**Table S5.** Associations between long-term residential PM_2.5_ and NO_2_ concentrations and risk of endometrial and ovarian cancers in the NIH-AARP Diet and Health Study, excluding women who were known to have moved between enrollment and 2012

**Table S6.** Associations between lagged long-term residential PM_2.5_ and NO_2_ concentrations and risk of endometrial and ovarian cancers in the NIH-AARP Diet and Health Study

**Table S7.** Cross-classified (common referent) associations between tertiles of long-term residential PM_2.5_ (µg/m^3^) and NO_2_ (ppb) concentrations and risk of endometrial and ovarian cancers in the NIH-AARP Diet and Health Study

**Figure S1.** Directed acyclic graph of the association between outdoor air pollution and ovarian cancer.

**Figure S2.** Directed acyclic graph of the association between outdoor air pollution and endometrial cancer.

Table S1. Baseline characteristics of NIH-AARP Diet and Health Study female participants included in the analyses of ovarian cancer, by historical outdoor PM_2.5_ levels at the residence**^a, b^**

| **Characteristics** | **All participants** | **PM_2.5_ concentration (1980-1984; μg/m^3^)** | | | |
| --- | --- | --- | --- | --- | --- |
|  |  | **Q1: 3.5-13.3** | **Q2: 13.4-15.6** | **Q3: 15.7-17.7** | **Q4: 17.8-27.0** |
| **Participants (N)** | 140,228 | 35,048 | 35,058 | 35,071 | 35,051 |
| **NO_2_, ppb (mean ± SD)^c^** | 15.9 ± 9.1 | 8.8 ± 3.7 | 12.8 ± 5.4 | 16.7 ± 6.21 | 25.3 ± 10.2 |
| **Age (mean ± SD)** | 61.7 ± 5.4 | 62.1 ± 5.4 | 61.6 ± 5.5 | 61.6 ± 5.4 | 61.7 ± 5.4 |
| **Age groups (%)** | | | | | |
| 50-54 years | 15.2 | 13.9 | 15.7 | 15.8 | 15.3 |
| 55-59 years | 23.4 | 21.9 | 24.0 | 23.9 | 23.9 |
| 60-64 years | 27.7 | 28.1 | 27.4 | 27.8 | 27.7 |
| 65-69 years | 30.3 | 32.5 | 29.5 | 29.4 | 29.8 |
| ≥ 70 years | 3.4 | 3.7 | 3.4 | 3.2 | 3.3 |
| **Race and ethnicity (%)** | | | | | |
| Non-Hispanic White | 89.7 | 93.6 | 92.1 | 90.6 | 82.7 |
| Non-Hispanic Black | 5.3 | 2.1 | 3.2 | 5.5 | 10.4 |
| Hispanic | 1.9 | 2.2 | 1.6 | 1.2 | 2.7 |
| Asian, PI, or AI/AK | 1.7 | 0.9 | 1.9 | 1.5 | 2.4 |
| Unknown | 1.4 | 1.2 | 1.2 | 1.3 | 1.8 |
| **Education level (%)** | | | | | |
| less than high school | 5.8 | 5.9 | 5.5 | 5.7 | 6.0 |
| completed high school | 25.0 | 24.6 | 24.2 | 26.1 | 25.2 |
| post high school | 10.4 | 11.4 | 10.6 | 10.0 | 9.4 |
| some college | 24.5 | 26.2 | 24.2 | 22.7 | 24.9 |
| college and postgraduate | 31.3 | 28.8 | 32.6 | 32.8 | 31.2 |
| unknown | 3.0 | 3.2 | 2.9 | 2.8 | 3.2 |
| **BMI (mean ± SD)** | 26.7 ± 5.7 | 26.4 ± 5.4 | 26.6 ± 5.6 | 26.8 ± 5.7 | 27.1 ± 5.9 |
| **BMI category (%) ^d^** | | | | | |
| Underweight and normal | 42.3 | 44.1 | 42.9 | 42.1 | 40.0 |
| Overweight | 32.0 | 32.4 | 31.9 | 32.0 | 31.7 |
| Obese | 22.4 | 20.2 | 21.9 | 22.6 | 24.8 |
| Unknown | 3.4 | 3.3 | 3.3 | 3.3 | 3.6 |
| **Postmenopausal status (%)** |  |  |  |  |  |
| Premenopausal | 4.9 | 4.4 | 5.2 | 5.0 | 5.0 |
| Postmenopausal | 94.7 | 95.2 | 94.5 | 94.6 | 94.6 |
| Unknown | 0.4 | 0.4 | 0.4 | 0.4 | 0.5 |
| **Smoking status (%)** | | | | | |
| Never smoker | 44.6 | 41.7 | 45.5 | 46.0 | 45.3 |
| Former smoker | 36.4 | 38.3 | 36.6 | 35.5 | 35.2 |
| Current smoker | 16.1 | 17.2 | 14.9 | 15.6 | 16.6 |
| Unknown | 2.9 | 2.9 | 2.9 | 2.9 | 3.0 |
| **State of residence (%)** | | | | | |
| California | 32.4 | 25.5 | 32.3 | 23.4 | 48.4 |
| Florida | 20.2 | 62.1 | 17.0 | 1.6 | 0.0 |
| Atlanta, Georgia | 2.8 | 0.0 | 0.2 | 3.1 | 7.7 |
| Louisiana | 3.4 | 2.3 | 6.4 | 4.8 | 0.0 |
| Detroit, Michigan | 5.2 | 0.4 | 3.5 | 9.2 | 7.8 |
| North Carolina | 8.2 | 3.7 | 11.5 | 14.6 | 2.9 |
| New Jersey | 13.0 | 4.4 | 20.9 | 21.0 | 5.7 |
| Pennsylvania | 14.9 | 1.7 | 8.2 | 22.1 | 27.5 |
| **Neighborhood Socioeconomic Deprivation Index Quintiles ^e^** | | | | | |
| Quintile 1 (least deprived) | - | 10.5 | 25.6 | 26.4 | 17.4 |
| Quintile 2 | - | 19.0 | 21.6 | 22.5 | 17.0 |
| Quintile 3 | - | 24.8 | 18.9 | 18.6 | 17.7 |
| Quintile 4 | - | 26.8 | 18.1 | 17.0 | 18.1 |
| Quintile 5 (most deprived) | - | 18.9 | 15.8 | 15.5 | 29.8 |
| a: Percentages may not add up to 100% due to rounding.  b: Abbreviations: PM: particulate matter; SD: standard deviation; BMI: body mass index.  c: Based on mean of annual values 1990 - 1994.  d: 4,713 women had missing BMI data in the ovarian cancer analysis group  e: Underweight and normal: BMI <25.0 kg/m^2^; overweight: 25.0-29.9 kg/m^2^; obese: ≥ 30.0 kg/m^2^. | | | | | |

Table S2. Baseline characteristics of NIH-AARP Diet and Health Study population included in the analyses of endometrial cancer, by historical ambient NO_2_ levels at the residence**^a, b^**

| **Characteristics** | **All participants** | **NO_2_ concentration (1990-1994; ppb)** | | | |
| --- | --- | --- | --- | --- | --- |
|  |  | **Q1: 0.8-9.5** | **Q2: 9.6-14.3** | **Q3: 14.4-20.3** | **Q4: 20.4-86.6** |
| **Participants (N)** | 116,380 | 29,095 | 29,095 | 29,095 | 29,095 |
| **PM_2.5_, µg/m^3^ (mean ± SD) ^c^** | 15.7 ± 3.2 | 15.4 ± 2.5 | 17.5 ± 2.6 | 19.8 ± 2.4 | 22.4 ± 2.8 |
| **Age (mean ± SD)** | 61.7 ± 5.5 | 61.9 ± 5.4 | 61.6 ± 5.5 | 61.6 ± 5.5 | 61.5 ± 5.5 |
| **Age groups (%)** | | | | | |
| 50-54 years | 15.8 | 14.5 | 16.6 | 16.1 | 16.3 |
| 55-59 years | 23.3 | 22.6 | 22.7 | 23.3 | 24.6 |
| 60-64 years | 27.2 | 27.4 | 27.2 | 27.0 | 27.0 |
| 65-69 years | 30.3 | 31.9 | 30.1 | 30.3 | 29.0 |
| ≥ 70 years | 3.4 | 3.6 | 3.4 | 3.4 | 3.2 |
| **Race and ethnicity (%)** | | | | | |
| Non-Hispanic White | 90.3 | 94.7 | 93.6 | 90.0 | 83.1 |
| Non-Hispanic Black | 4.7 | 2.6 | 2.8 | 4.9 | 8.7 |
| Hispanic | 1.9 | 0.9 | 1.5 | 2.0 | 3.0 |
| Asian, PI, or AI/AK Native | 1.8 | 0.9 | 1.1 | 1.7 | 3.5 |
| Unknown | 1.3 | 1.0 | 1.0 | 1.4 | 1.7 |
| **Education level (%)** | | | | | |
| Less Than 8 years of Education | 5.5 | 6.56 | 4.87 | 4.99 | 5.45 |
| 8-11 Years of Education | 24.7 | 25.98 | 24.39 | 25.18 | 23.18 |
| 12 years of Education | 10.0 | 11.31 | 10.51 | 9.66 | 8.54 |
| Post-High School Training | 24.0 | 23.17 | 23.78 | 23.13 | 25.96 |
| Some College | 33.0 | 30.07 | 33.73 | 34.45 | 33.73 |
| College graduate | 2.8 | 2.9 | 2.72 | 2.6 | 3.13 |
| **BMI (mean ± SD)** | 26.7 ± 5.7 | 26.5 ± 5.5 | 26.4 ± 5.6 | 26.7± 5.7 | 27.0 ± 5.9 |
| **BMI category (%) ^d^** | | | | | |
| Underweight and normal | 43.3 | 43.5 | 44.9 | 43.3 | 41.5 |
| Overweight | 31.5 | 32.3 | 31.5 | 31.4 | 31.0 |
| Obese | 21.8 | 21.0 | 20.4 | 22.2 | 23.6 |
| Unknown | 3.4 | 3.3 | 3.3 | 3.2 | 3.9 |
| **Postmenopausal status (%)** |  |  |  |  |  |
| Premenopausal | 6.2 | 5.8 | 6.6 | 6.2 | 6.2 |
| Postmenopausal | 93.3 | 93.8 | 92.9 | 93.3 | 93.2 |
| Unknown | 0.5 | 0.4 | 0.5 | 0.4 | 0.6 |
| **Smoking status (%)** | | | | | |
| Never smoker | 43.8 | 44.5 | 43.1 | 43.9 | 43.6 |
| Former smoker | 36.8 | 36.1 | 37.5 | 36.9 | 36.6 |
| Current smoker | 16.5 | 16.6 | 16.6 | 16.3 | 16.7 |
| Unknown | 2.9 | 2.9 | 2.9 | 2.9 | 3.1 |
| **State of residence (%)** | | | | | |
| California | 31.8 | 16.5 | 17.5 | 30.4 | 62.7 |
| Florida | 20.0 | 40.0 | 31.7 | 8.1 | 0.3 |
| Atlanta, Georgia | 2.6 | 1.5 | 4.6 | 3.5 | 0.8 |
| Louisiana | 2.7 | 5.9 | 2.4 | 2.2 | 0.3 |
| Detroit, Michigan | 5.3 | 3.0 | 6.5 | 8.5 | 3.2 |
| North Carolina | 7.5 | 18.5 | 7.3 | 3.5 | 0.5 |
| New Jersey | 14.6 | 6.3 | 14.9 | 18.8 | 18.4 |
| Pennsylvania | 15.5 | 8.3 | 15.2 | 24.9 | 13.7 |
| **Neighborhood Socioeconomic Deprivation Index Quintiles** | | | | | |
| Quintile 1 (least deprived) | - | 10.9 | 23.0 | 24.6 | 18.9 |
| Quintile 2 | - | 15.8 | 22.0 | 21.6 | 19.9 |
| Quintile 3 | - | 27.9 | 18.8 | 17.1 | 16.1 |
| Quintile 4 | - | 21.2 | 20.6 | 18.6 | 17.9 |
| Quintile 5 (most deprived) | - | 24.2 | 15.7 | 18.2 | 27.2 |
| a: Percentages may not add up to 100% due to rounding.  b: Abbreviations: NO: nitrogen dioxide; SD: standard deviation; BMI: body mass index; ppb: parts per billion  c: Based on mean of annual values during 1980-1984.  d: Underweight and normal: BMI <25.0 kg/m^2^; overweight: 25.0-29.9 kg/m^2^; obese: ≥30.0 kg/m^2^. | | | | | |

Table S3. Baseline characteristics of NIH-AARP Diet and Health Study population included in the analyses of ovarian cancer, by historical ambient NO_2_ levels at the residence**^a, b^**

| **Characteristics** | **All participants** | **NO_2_ concentration (1990-1994; ppb)** | | | |
| --- | --- | --- | --- | --- | --- |
|  |  | **Q1: 0.9-9.4** | **Q2: 9.5-14.2** | **Q3: 14.3-20.2** | **Q4: 20.3-86.6** |
| **Participants (N)** | 140,228 | 35,063 | 35,051 | 35,057 | 35,057 |
| **PM_2.5_, µg/m^3^ (mean ± SD) ^c^** | 18.8 ± 3.7 | 15.4 ± 2.6 | 17.5 ± 2.6 | 19.7 ± 2.4 | 22.4 ± 2.8 |
| **Age (mean ± SD)** | 61.7 ± 5.4 | 61.9 ± 5.4 | 61.7 ± 5.5 | 61.7 ± 5.5 | 61.6 ± 5.4 |
| **Age groups (%)** | | | | | |
| 50-54 years | 15.2 | 14.0 | 15.8 | 15.4 | 15.4 |
| 55-59 years | 23.4 | 23.0 | 23.0 | 23.3 | 24.5 |
| 60-64 years | 27.7 | 28.1 | 27.7 | 27.5 | 27.6 |
| 65-69 years | 30.3 | 31.4 | 30.1 | 30.4 | 29.3 |
| ≥ 70 years | 3.4 | 3.6 | 3.4 | 3.4 | 3.2 |
| **Race and ethnicity (%)** | | | | | |
| Non-Hispanic White | 89.7 | 94.4 | 93.1 | 89.2 | 82.3 |
| Non-Hispanic Black | 5.3 | 2.8 | 3.1 | 5.7 | 9.6 |
| Hispanic | 1.9 | 0.9 | 1.6 | 2.0 | 3.2 |
| Asian, PI, or AI/AK Native | 1.7 | 0.8 | 1.1 | 1.6 | 3.1 |
| Unknown | 1.4 | 1.1 | 1.1 | 1.5 | 1.9 |
| **Education level (%)** | | | | | |
| Less Than 8 years of Education | 5.8 | 7.0 | 5.2 | 5.3 | 5.7 |
| 8-11 Years of Education | 25.0 | 26.6 | 24.8 | 25.4 | 23.3 |
| 12 years of Education | 10.4 | 11.8 | 10.9 | 10.0 | 8.8 |
| Post-High School Training | 24.5 | 23.4 | 24.1 | 23.9 | 26.6 |
| Some College | 31.3 | 28.3 | 32.0 | 32.7 | 32.3 |
| College graduate | 3.0 | 3.1 | 2.9 | 2.8 | 3.3 |
| **BMI (mean ± SD)** | 26.7 ± 5.7 | 25.6 ± 5.5 | 26.5 ± 5.5 | 26.8 ± 5.7 | 27.1 ± 5.9 |
| **BMI category (%) ^d^** | | | | | |
| Underweight and normal | 42.3 | 42.3 | 43.9 | 42.2 | 40.5 |
| Overweight | 32.0 | 32.9 | 32.1 | 31.7 | 31.3 |
| Obese | 22.4 | 21.6 | 20.8 | 22.8 | 24.4 |
| Unknown | 3.4 | 3.2 | 3.2 | 3.2 | 3.8 |
| **Postmenopausal status (%)** | | | | | |
| Premenopausal \| 4.9 \| 4.4 \| 5.2 \| 5.0 \| 5.0; Postmenopausal \| 94.7 \| 95.3 \| 94.4 \| 94.7 \| 94.6; Unknown \| 0.4 \| 0.4 \| 0.4 \| 0.4 \| 0.5 | | | | | |
| **Smoking status (%)** | | | | | |
| Never smoker | 44.6 | 45.7 | 44.0 | 44.6 | 44.1 |
| Former smoker | 36.4 | 35.4 | 37.0 | 36.6 | 36.5 |
| Current smoker | 16.1 | 16.0 | 16.2 | 16.0 | 16.2 |
| Unknown | 2.9 | 2.8 | 2.9 | 2.9 | 3.2 |
| **State of residence (%)** | | | | | |
| California | 32.4 | 16.7 | 17.6 | 31.0 | 64.4 |
| Florida | 20.2 | 39.2 | 32.5 | 8.7 | 0.3 |
| Georgia | 2.8 | 1.5 | 4.9 | 3.8 | 0.9 |
| Louisiana | 3.4 | 6.9 | 3.2 | 3.0 | 0.4 |
| Michigan | 5.2 | 2.8 | 6.1 | 8.5 | 3.4 |
| North Carolina | 8.2 | 19.9 | 8.3 | 4.0 | 0.6 |
| New Jersey | 13.0 | 5.5 | 13.0 | 17.0 | 16.6 |
| Pennsylvania | 14.9 | 7.5 | 14.6 | 23.9 | 13.4 |
| **Neighborhood Socioeconomic Deprivation Index Quintiles ^d^** | | | | | |
| Q1 (least deprived) | - | 11.0 | 23.6 | 25.6 | 19.8 |
| Q2 | - | 16.0 | 21.7 | 21.7 | 20.6 |
| Q3 | - | 22.5 | 21.6 | 18.2 | 17.8 |
| Q4 | - | 28.8 | 18.3 | 17.0 | 15.9 |
| Q5 (most deprived) | - | 21.8 | 14.8 | 17.5 | 26.0 |
| a: Percentages may not add up to 100% due to rounding.  b: Abbreviations: NO: nitrogen dioxide; SD: standard deviation; BMI: body mass index; ppb: parts per billion  c: Based on mean of annual values during 1980-1984.  d: Underweight and normal: BMI <25.0 kg/m^2^; overweight: 25.0-29.9 kg/m^2^; obese: ≥30.0 kg/m^2^. | | | | | |

Table S4. Associations between 5-year historical average residential PM_2.5_ (µg/m^3^) and NO_2_ (ppb) concentrations and risk of endometrial and ovarian cancers in the NIH-AARP Diet and Health Study^a^

| **1980-1984** | | | | | | | |
| --- | --- | --- | --- | --- | --- | --- | --- |
| **Endometrial Cancer** | | | | **Ovarian Cancer** | | | |
| **PM_2.5_** | **Cases** | **HR (95% CI)** |  |  | **Cases** | **HR (95% CI)** |  |
|  |  | **Model 1^b^** | **Model 2^c^** |  |  | **Model 1^b^** | **Model 2^c^** |
| Per 5 μg/m^3^ | 3,088 | 1.01 (0.95-1.07) | - | Per 5 μg/m^3^ | 1,582 | 0.97 (0.90-1.05) | - |
| Q1 | 731 | 1.00 (referent) | - | Q1 | 402 | 1.00 (referent) | - |
| Q2 | 751 | 0.92 (0.82-1.02) | - | Q2 | 390 | 0.94 (0.81-1.09) | - |
| Q3 | 770 | 0.92 (0.82-1.04) | - | Q3 | 400 | 0.96 (0.82-1.14) | - |
| Q4 | 836 | 1.00 (0.89-1.13) | - | Q4 | 390 | 0.94 (0.79-1.11) | - |
| *p* for trend |  | 0.76 | - |  |  | 0.56 | - |
| **1985-1989** | | | | | | | |
| **Endometrial Cancer** | | | | **Ovarian Cancer** | | | |
| **PM_2.5_** | **Cases** | **HR (95% CI)** |  |  | **Cases** | **HR (95% CI)** |  |
|  |  | **Model 1^b^** | **Model 2^c^** |  |  | **Model 1^b^** | **Model 2^c^** |
| Per 5 μg/m^3^ | 3,088 | 1.01 (0.95-1.07) | - | Per 5 μg/m^3^ | 1,582 | 0.97 (0.89-1.05) | - |
| Q1 | 732 | 1.00 (referent) | - | Q1 | 396 | 1.00 (referent) | - |
| Q2 | 750 | 0.90 (0.80-1.01) | - | Q2 | 390 | 0.95 (0.82-1.11) | - |
| Q3 | 779 | 0.92 (0.82-1.04) | - | Q3 | 396 | 0.98 (0.82-1.15) | - |
| Q4 | 827 | 0.98 (0.86-1.11) | - | Q4 | 400 | 0.98 (0.83-1.17) | - |
| *p* for trend |  | 0.99 | - |  |  | 0.94 | - |
| **1990-1994** | | | | | | | |
| **Endometrial Cancer** | | | | **Ovarian Cancer** | | | |
| **PM_2.5_** | **Cases** | **HR (95% CI)** | **HR (95% CI)** |  | **Cases** | **HR (95% CI)** | **HR (95% CI)** |
|  |  | **Model 1^b^** | **Model 2^c^** |  |  | **Model 1^b^** | **Model 2^c^** |
| Per 5 μg/m^3^ | 3,088 | 1.01 (0.94-1.08) | 0.99 (0.89-1.09) | Per 5 μg/m^3^ | 1,582 | 0.96 (0.88-1.05) | 0.97 (0.84-1.11) |
| Q1 | 733 | 1.00 (referent) | 1.00 (referent) | Q1 | 395 | 1.00 (referent) | 1.00 (referent) |
| Q2 | 751 | 0.89 (0.79-1.00) | 0.88 (0.78-0.99) | Q2 | 396 | 0.96 (0.82-1.13) | 0.98 (0.83-1.16) |
| Q3 | 766 | 0.89 (0.79-1.00) | 0.87 (0.75-1.02) | Q3 | 389 | 0.95 (0.80-1.14) | 0.98 (0.80-1.21) |
| Q4 | 838 | 0.98 (0.86-1.11) | 0.95 (0.81-1.13) | Q4 | 402 | 0.97 (0.82-1.16) | 1.00 (0.79-1.26) |
| *p* for trend |  | 0.94 | 0.82 |  |  | 0.80 | 0.95 |
| **NO_2_** | **Cases** | **HR (95% CI)** | **HR (95% CI)** |  | **Cases** | **HR (95% CI)** | **HR (95% CI)** |
|  |  | **Model 1^b^** | **Model 2^c^** |  |  | **Model 1^b^** | **Model 2^c^** |
| Per 5 ppb | 3,088 | 1.01 (0.98-1.03) | 1.01 (0.98-1.03) | Per 5 ppb | 1,582 | 0.99 (0.96-1.02) | 1.00 (0.95-1.05) |
| Q1 | 733 | 1.00 (referent) | 1.00 (referent) | Q1 | 407 | 1.00 (referent) | 1.00 (referent) |
| Q2 | 739 | 0.98 (0.89-1.09) | 1.01 (0.91-1.13) | Q2 | 371 | 0.91 (0.79-1.05) | 0.91 (0.78-1.06) |
| Q3 | 786 | 0.99 (0.89-1.11) | 1.02 (0.90-1.16) | Q3 | 405 | 0.97 (0.83-1.13) | 0.97 (0.82-1.16) |
| Q4 | 830 | 1.02 (0.91-1.14) | 1.04 (0.89-1.22) | Q4 | 399 | 0.94 (0.80-1.11) | 0.94 (0.75-1.17) |
| *p* for trend |  | 0.70 | 0.64 |  |  | 0.66 | 0.71 |

a: Abbreviations: PM: particulate matter; NO: nitrogen dioxide; HR: hazard ratio; CI: confidence interval

b: Adjusted for age, race and ethnicity, smoking status and intensity, neighborhood socioeconomic deprivation index, body mass index (endometrial analyses only), and state of residence.

c: Adjusted for Model 1 covariates and mutually adjusted for either NO_2_ or PM_2.5._ NO_2_ estimates were available starting in 1990; therefore, models based on data for 1980-1984 and 1985-1989 do not include NO_2_.

Table S5. Associations between long-term residential PM_2.5_ and NO_2_ concentrations and risk of endometrial and ovarian cancers in the NIH-AARP Diet and Health Study, excluding women who were known to have moved between enrollment and 2012^a^

| **Endometrial Cancer** | | | |  |  | **Ovarian Cancer** | |
| --- | --- | --- | --- | --- | --- | --- | --- |
| **PM_2.5_** | **Cases** | **HR (95% CI)**  **Model 1 ^b^** | **HR (95% CI)**  **Model 2^c^** |  | **Cases** | **HR (95% CI)**  **Model 1 ^b^** | **HR (95% CI)**  **Model 2^c^** |
| Per 5 μg/m^3^ | 1,496 | 0.98 (0.88-1.09) | 0.89 (0.76-1.05) | Per 5 μg/m^3^ | 786 | 1.01 (0.87-1.18) | 1.01 (0.81-1.26) |
| Q1 | 334 | 1.00 (referent) | 1.00 (referent) | Q1 | 190 | 1.00 (referent) | 1.00 (referent) |
| Q2 | 408 | 0.97 (0.82-1.14) | 0.95 (0.79-1.13) | Q2 | 207 | 0.97 (0.78-1.21) | 0.97 (0.77-1.23) |
| Q3 | 395 | 0.97 (0.81-1.16) | 0.93 (0.75-1.14) | Q3 | 200 | 1.01 (0.79-1.29) | 1.00 (0.75-1.32) |
| Q4 | 359 | 0.95 (0.79-1.14) | 0.89 (0.71-1.12) | Q4 | 189 | 1.03 (0.80-1.32) | 1.01 (0.74-1.38) |
| *p* for trend | | 0.66 | 0.40 |  |  | 0.53 | 0.63 |
|  | | | |  |  |  |  |
| **NO_2_** | **Cases** | **HR (95% CI)**  **Model 1 ^b^** | **HR (95% CI)**  **Model 2^c^** |  | **Cases** | **HR (95% CI)**  **Model 1 ^b^** | **HR (95% CI)**  **Model 2^c^** |
| Per 5 ppb | 1.496 | 1.02 (0.98-1.06) | 1.05 (0.99-1.17) | Per 5 ppb | 786 | 1.00 (0.94-1.07) | 1.00 (0.92-1.09) |
| Q1 | 393 | 1.00 (referent) | 1.00 (referent) | Q1 | 227 | 1.00 (referent) | 1.00 (referent) |
| Q2 | 363 | 1.04 (0.90-120) | 1.06 (0.91-1.24) | Q2 | 183 | 0.95 (0.77-1.16) | 0.95 (0.77-1.17) |
| Q3 | 374 | 1.01 (0.87-1.18) | 1.05 (0.88-1.25) | Q3 | 196 | 1.02 (0.82-1.26) | 1.02 (0.80-1.29) |
| Q4 | 366 | 1.04 (0.88-1.22) | 1.10 (0.89-1.36) | Q4 | 180 | 1.03 (0.82-1.30) | 1.02 (0.76-1.37) |
| *p* for trend | | 0.60 | 0.29 |  |  | 0.81 | 0.90 |

a: Abbreviations: PM: particulate matter; NO_2_: nitrogen dioxide; HR: hazard ratio; CI: confidence interval, μg/m^3^: micrograms per cubic meter; ppb: parts per billion.
b: Adjusted for age, race and ethnicity, smoking status and intensity, neighborhood socioeconomic deprivation index, body mass index (endometrial cancer only), and state of residence.
c: Adjusted for Model 1 covariates and mutually adjusted for either NO_2_ or PM_2.5_.

Table S5. Associations between lagged long-term residential PM_2.5_ and NO_2_ concentrations and risk of endometrial and ovarian cancers in the NIH-AARP Diet and Health Study^a^

| **Endometrial Cancer** | | | | | | | **Ovarian Cancer** | | | | | |
| --- | --- | --- | --- | --- | --- | --- | --- | --- | --- | --- | --- | --- |
| **PM_2.5_** | **Cases** | **HR (95% CI)** |  | **Cases** | **HR (95% CI)** | |  | **Cases** | **HR (95% CI)** | | **Cases** | **HR (95% CI)** |
|  |  | **Model 1^b^** |  |  | **Model 2^d^** | |  |  | **Model 1^b^** |  |  | **Model 2^d^** |
| Per 5 μg/m^3^ | 3,088 | 1.00 (0.94-1.07) | Per 5 μg/m^3^ | 3,088 | 0.98 (0.89-1.09) | | Per 5 μg/m^3^ | 1,582 | 0.96 (0.88-1.05) | Per 5 μg/m^3^ | 1,582 | 0.92 (0.80-1.06) |
| Q1 | 739 | 1.00 (referent) | Q1 | 738 | 1.00 (referent) | | Q1 | 406 | 1.00 (referent) | Q1 | 412 | 1.00 (referent) |
| Q2 | 751 | 0.91 (0.81-1.02) | Q2 | 729 | 0.89 (0.78-1.01) | | Q2 | 382 | 0.92 (0.79-1.08) | Q2 | 391 | 0.90 (0.76-1.07) |
| Q3 | 771 | 0.93 (0.82-1.06) | Q3 | 803 | 0.99 (0.86-1.16) | | Q3 | 402 | 0.98 (0.83-1.17) | Q3 | 386 | 0.89 (0.73-1.10) |
| Q4 | 827 | 0.99 (0.87-1.12) | Q4 | 818 | 1.01 (0.85-1.19) | | Q4 | 392 | 0.93 (0.78-1.10) | Q4 | 393 | 0.87 (0.69-1.09) |
| *p* for trend | | 0.90 |  | | 0.90 | |  |  | 0.56 |  |  | 0.3 |
|  |  |  |  |  |  |  |  |  |  |  |  |  |
| **NO_2_** | **Cases** | **HR (95% CI)** |  | **Cases** | **HR (95% CI)** | |  | **Cases** | **HR (95% CI)** | | **Cases** | **HR (95% CI)** |
|  |  | **Model 1^c^** |  |  | **Model 2^e^** | |  |  | **Model 1^c^** |  |  | **Model 2^e^** |
| Per 5 ppb | 3,088 | 1.01 (0.98-1.04) | Per 5 ppb | 3,088 | 1.01 (0.97-1.06) | | Per 5 ppb | 1,582 | 0.99 (0.95-1.03) | Per 5 ppb | 1,582 | 1.01 (0.95-1.08) |
| Q1 | 751 | 1.00 (referent) | Q1 | 751 | 1.00 (referent) | | Q1 | 409 | 1.00 (referent) | Q1 | 409 | 1.00 (referent) |
| Q2 | 730 | 0.96 (0.87-1.07) | Q2 | 730 | 0.97 (0.87-1.08) | | Q2 | 371 | 0.92 (0.79-1.05) | Q2 | 371 | 0.94 (0.81-1.10) |
| Q3 | 786 | 0.98 (0.88-1.09) | Q3 | 786 | 0.98 (0.86-1.11) | | Q3 | 412 | 1.00 (0.86-1.16) | Q3 | 412 | 1.05 (0.89-1.25) |
| Q4 | 821 | 0.99 (0.88-1.11) | Q4 | 821 | 0.96 (0.82-1.11) | | Q4 | 390 | 0.94 (0.80-1.11) | Q4 | 390 | 1.02 (0.82-1.25) |
| *p* for trend | | 0.90 |  | | 0.81 | |  |  | 0.65 |  |  | 0.84 |

a: Abbreviations: PM: particulate matter; NO_2_: nitrogen dioxide; HR: hazard ratio; CI: confidence interval, μg/m^3^: micrograms per cubic meter; ppb: parts per billion.
b: PM_2.5_ 5-year average lagged 10 years; adjusted for age, race and ethnicity, smoking status and intensity, neighborhood socioeconomic deprivation index, body mass index (endometrial cancer only), and state of residence.

c: NO_2_ 5-year average lagged 5 years; adjusted for age, race and ethnicity, smoking status and intensity, neighborhood socioeconomic deprivation index, body mass index (endometrial cancer only), and state of residence.

d: PM_2.5_ 5-year average lagged 5 years mutually adjusted for Model 1 covariates and 5-year average 5-year lagged NO_2_

e: NO_2_ 5-year average lagged 5 years mutually adjusted for Model 1 covariates and 5-year average 5-year lagged PM_2.5_

Table S7. Cross-classified (common referent) associations between tertiles of long-term residential PM_2.5_ (µg/m^3^) and NO_2_ (ppb) concentrations and risk of endometrial and ovarian cancers in the NIH-AARP Diet and Health Study ^a,b^

| **ENDOMETRIAL CANCER (n=116,380)** | | | | | | |
| --- | --- | --- | --- | --- | --- | --- |
|  | **NO_2_ (ppb)** | | | | | |
|  | **T1: 0.9–11.0** | | **T2: 11.1–18.0** | | **T3: 18.1–86.6** | |
| **PM_2.5_ (μg/m^3^)** | **Cases (n)** | **HR (95%CI)^b^** | **Cases (n)** | **HR (95%CI)** | **Cases (n)** | **HR (95%CI)** |
| **T1: 3.5–14.1** | 669 | 1.00 (referent) | 258 | 0.95 (0.82-1.09) | 16 | 0.56 (0.33-0.95) |
| **T2: 14.2–17.0** | 285 | 0.88 (0.75-1.03) | 462 | 0.90 (0.78-1.02) | 298 | 0.91 (0.78-1.06) |
| **T3: 17.1–27.0** | 22 | 0.93 (0.65-1.32) | 297 | 0.97 (0.83-1.14) | 771 | 1.00 (0.88-1.13) |
|  | | | | | | |
| **OVARIAN CANCER (n=140,228)** | | | | | | |
|  | **NO_2_ (ppb)** | | | | | |
|  | **T1: 0.9–10.9** | | **T2: 10.10–17.9** | | **T3: 18.0–86.6** | |
| **PM_2.5_ (μg/m^3^)** | **Cases (n)** | **HR (95%CI)** | **Cases (n)** | **HR (95%CI)** | **Cases (n)** | **HR (95%CI)** |
| **T1: 3.5–14.1** | 345 | 1.00 (referent) | 159 | 1.04 (0.86-1.26) | 7 | 0.50 (0.24-1.06) |
| **T2: 14.2–17.0** | 158 | 1.05 (0.85-1.30) | 222 | 0.96 (0.80-1.16) | 167 | 1.03 (0.84-1.27) |
| **T3: 17.1–27.0** | 16 | 0.83 (0.49-1.41) | 146 | 0.95 (0.75-1.20) | 362 | 1.02 (0.86-1.22) |
| a: Abbreviations: PM: particulate matter; NO: nitrogen dioxide; HR: hazard ratio; CI: confidence interval | | | | | | |
| b: Models are adjusted for age, race and ethnicity, smoking status and intensity, neighborhood socioeconomic deprivation index, body mass index (endometrial analyses only), and state of residence. | | | | | | |

Figure S1. Directed acyclic graph of the association between outdoor air pollution and ovarian cancer.

Figure S2. Directed acyclic graph of the association between outdoor air pollution and endometrial cancer.
